# Cyclic Derivatives of the Chemerin C-Terminus as Metabolically Stable Agonists at the Chemokine-like Receptor 1 for Cancer Treatment

**DOI:** 10.3390/cancers13153788

**Published:** 2021-07-27

**Authors:** Tobias F. Fischer, Anne S. Czerniak, Tina Weiß, Tristan Zellmann, Lina Zielke, Sylvia Els-Heindl, Annette G. Beck-Sickinger

**Affiliations:** Institute of Biochemistry, Leipzig University, Brüderstraße 34, 04103 Leipzig, Germany; tobias.fischer@uni-leipzig.de (T.F.F.); anne.czerniak@uni-leipzig.de (A.S.C.); tina.weiss@uni-leipzig.de (T.W.); tristan.zellmann@googlemail.com (T.Z.); lz32mani@studserv.uni-leipzig.de (L.Z.); sylvia.els@uni-leipzig.de (S.E.-H.)

**Keywords:** chemerin, CMKLR1, peptides, peptide–drug conjugates, GPCR

## Abstract

**Simple Summary:**

The innate immune system is a key player in the fight against tumors and metastasis. Chemerin induces the migration of immune cells towards sites of inflammation and is crucial for recruiting natural killer cells towards cancerous tissue. Several cancer types are able to evade the immune system by downregulating levels of chemerin in their environment, which is often associated with decreased patient survival. Treatment with chemerin could counteract this strategy and thus be beneficial for cancer immunotherapy. Here, we report on the design of small synthetic peptides derived from chemerin that are biologically active and stable in blood plasma. They could be used to specifically deliver chemotherapeutics to tumors that express the chemokine-like receptor 1 (CMKLR1) or support the immune system in fighting cancer. Thus, they represent promising cancer therapeutics.

**Abstract:**

Chemerin is a small chemotactic protein and a modulator of the innate immune system. Its activity is mainly mediated by the chemokine-like receptor 1 (CMKLR1), a receptor expressed by natural killer cells, dendritic cells, and macrophages. Downregulation of chemerin is part of the immune evasion strategy exploited by several cancer types, including melanoma, breast cancer, and hepatocellular carcinoma. Administration of chemerin can potentially counteract these effects, but synthetically accessible, metabolically stable analogs are required. Other tumors display overexpression of CMKLR1, offering a potential entry point for targeted delivery of chemotherapeutics. Here, we present cyclic derivatives of the chemerin C-terminus (chemerin-9), the minimal activation sequence of chemerin. Chemerin-9 derivatives that were cyclized through positions four and nine retained activity while displaying full stability in blood plasma for more than 24 h. Therefore, these peptides could be used as a drug shuttle system to target cancer cells as demonstrated here by methotrexate conjugates.

## 1. Introduction

The early immune response mainly relies on the innate immune system, the first line of defense against infections and tumors. By mobilizing immune cells, soluble chemoattractants play an important role in this mechanism [1]. Consequently, dysregulation of these chemoattractants has severe consequences such as chronic inflammation on the one hand, or a diminished immune response on the other.

Chemerin, identified as the natural ligand of the chemokine-like receptor 1 (CMKLR1) by Wittamer et al., is a small chemoattractant protein and acts as an adipokine [2]. It is expressed as 163 amino acid preprochemerin and secreted as prochemerin after truncation of a 20 amino acid N-terminal signal peptide [2]. Primary sources of chemerin are adipose tissue, skin, and liver [3,4]. C-terminal cleavage by different proteases of the inflammation and coagulation cascades yields active chemerin species, which are named according to their last, C-terminal amino acid [5,6,7,8]. ChemerinS157 and ChemerinF156 are the active proteins, and further truncations result in inactive species [9]. The agonistic properties of chemerin are preserved in the C-terminus. Indeed, the C-terminal nine amino acids of ChemerinS157 (named chemerin-9) are sufficient for receptor activation, displaying only a two-fold loss of activity compared to the full-length protein [9]. The C-terminus of chemerin is folded into a loop structure and interacts with the extracellular loops of CMKLR1 to activate the receptor [10]. This interaction is predominantly mediated by the aromatic residues of the ligand [9].

CMKLR1 is a G protein-coupled receptor (GPCR), which is mainly expressed by adipocytes [3] and various leukocytes, i.e., natural killer (NK) cells, plasmacytoid dendritic cells, and macrophages [2,11,12]. Activation of CMKLR1 induces the migration of these immune cells towards sites of inflammation and evokes the release of further inflammatory cytokines such as interleukin-2, interleukin-8, and tumor necrosis factor-α from human chondrocytes [13]. On the other hand, CMKLR1 activation by chemerin promotes the migration and invasion of gastric and oesophageal squamous cancer cells [14,15].

Two additional GPCR have been identified as binding partners for chemerin. The C-C motif chemokine receptor-like 2 (CCRL2) binds chemerin, but no intracellular signaling response has been linked to this receptor so far [16,17]. CCRL2 seems to be important for the formation of a stable chemerin gradient, presenting chemerin to neighboring cells [16]. GPR1 is the closest homolog of CMKLR1, and the second signaling receptor activated by chemerin [18]. Activation of GPR1 does not lead to G protein signaling but induces recruitment of arrestin3 to the receptor [17].

Several studies have suggested a therapeutic potential for chemerin as part of an immune-supporting strategy in the treatment of cancer [19]. Here, we describe the development of small cyclic derivatives of chemerin-9. These peptides are fully stable in blood plasma for at least 24 h and thus overcome the main limitation of the linear parent peptide. They are highly constrained yet highly active and thus represent promising new therapeutic modalities to directly counteract tumor-induced immune evasion or by shuttling cytotoxic cargoes into tumor cells that express CMKLR1 as recently shown for prostate cancer cells [20].

## 2. Materials and Methods

### 2.1. Materials

Peptide Synthesis: Fmoc-protected amino acids were purchased from ORPEGEN (Heidelberg, Germany). Peptide resins, 1-hydroxy benzotriazole (HOBt), diiodomethane, ethanedithiol (EDT), diethyl ether, and trifluoracetic acid (TFA) were obtained from Merck (Darmstadt, Germany). N,N’-diisopropyl carbodiimide (DIC) and 2-cyano-2-(hydroxyimino) acetic acid ethyl ester (oxyma) were purchased from Iris Biotech (Marktredwitz, Germany). Dimethylformamide (DMF) and dichloromethane (DCM) were purchased from Biosolve (Valkenswaard, Netherlands), acetonitrile (ACN) was obtained from VWR (Darmstadt, Germany), and tetrahydrofuran (THF) was purchased from Grüssing (Filsum, Germany). O-(7-Azabenzotriazol-1-yl)-N,N,N,N-tetramethyluronium hexafluorophosphate (HATU), tris(2-carboxyethyl)phosphine hydrochloride (TCEP), *N,N*-diisopropylethylamine (DIPEA), piperidine, and thioanisole were obtained from Sigma-Aldrich (St. Louis, MO, USA). 6-Carboxytetramethylrhodamine (Tam) was purchased from emp biotech (Berlin, Germany). Triethylamine (Et_3_N) was purchased from Thermo Fisher Scientific (Waltham, USA). Methotrexate (MTX) was purchased from Absource Diagnostics GmbH (München, Deutschland).

Cell Culture: Cell culture media (Dulbecco’s Modified Eagle’s Medium (DMEM), Ham’s F12, MCDB131), as well as trypsin-EDTA, Dulbecco’s Phosphate-Buffered Saline (DPBS), and Hank’s Balanced Salt Solution (HBSS), were obtained from Lonza (Basel, Switzerland). Fetal bovine serum (FBS) was obtained from Biochrom GmbH (Berlin, Germany). Hygromycin B was purchased from Invivogen (Toulouse, France), and Opti-MEM was obtained from Life Technologies (Basel, Switzerland). LipofectamineTM 2000 was obtained from Invitrogen (Carlsbad, CA, USA). MetafecteneProTM was received from Biontex Laboratories GmbH (München, Germany). Coelenterazine H was purchased from DiscoverX (Fremont, CA, USA). Hoechst33342 nuclear stain was obtained from Sigma-Aldrich (St. Louis, MO, USA). Bovine arrestin-3 was fused to Rluc8 and cloned into the pcDNA3 vector for BRET studies. Pluronic and Fluo-2 AM were obtained from Abcam (Cambridge, UK). Probenecid was purchased from Sigma-Aldrich (St. Louis, MO, USA). In Vitro Toxicology Assay Kit, Resazurin based was purchased from Sigma-Aldrich (St. Louis, MO, USA). The plasmid coding for the chimeric G protein GαΔ6qi4myr was kindly provided by E. Kostenis, Rheinische Friedrich-Wilhelms-Universität, Bonn, Germany.

### 2.2. Peptide Synthesis

All peptides were synthesized using an orthogonal 9-fluorenylmethoxycarbonyl/tert-butyl (Fmoc/tBu) solid-phase peptide synthesis strategy. Standard synthesis of all peptides was performed on a Syro II peptide synthesizer (MultiSynTech, Bochum, Germany) on a scale of 15 µmol. Peptides were synthesized on a Wang resin or a 2-chlorotrityl chloride resin to obtain a C-terminal acid. Coupling reactions were performed twice with 8 equiv. of the respective, Fmoc-protected amino acid, which was activated in situ with equimolar amounts of oxyma and DIC in DMF for 30 min. Fmoc-deprotection was achieved by incubation with 40% piperidine in DMF (*v*/*v*) for 3 min and 20% piperidine in DMF (*v*/*v*) for 10 min. N-terminal Tam-labeling of the peptides used in stability assays was performed manually by reaction with 2 equiv. Tam, 1.9 equiv. HATU, 2 equiv. DIPEA in DMF for 2 h at room temperature (rt). N-terminal labeling with MTX for peptides used in resazurin assays was performed manually by reaction with 5 equiv. Mtx, 5 equiv. HOBt, 5 equiv. DIC in DMF for 3 h at rt. Acetylation of lysine side chains was achieved by incubation with acetic anhydride, N,N-diisopropylethylamine (DIPEA), and DMF (1:1:38) twice for 10 min at rt.

All peptides were cleaved by incubation with 90% TFA, 7% thioanisole, 3% ethanedithiol (*v*/*v*/*v*) for 3 h at rt. After full cleavage, the crude peptides were precipitated in cold diethyl ether/hexane at −20 °C for at least 3 h, washed with diethyl ether, and collected by centrifugation. Disulfide-bridged peptides were dissolved in refolding buffer (20% ACN, 0.15 M NaCl, 25 mM Tris, pH 7.7) and incubated at rt for 72 h for cyclization.

Thioacetal bridged peptides were generated from the corresponding purified, linear peptides, as described previously [21]. Briefly, the peptide was dissolved in THF/H_2_O (1:2) in the presence of 3 equiv. K_2_CO_3_, 3 equiv. TCEP, and 20 equiv. Et_3_N. This solution was added stepwise to a solution of 20 equiv. Ch_2_I_2_ in THF. The reaction was completed after shaking at rt for 12 h.

All peptides were purified by RP-HPLC on a Kinetex 5 µm XB-C_18_ 100 Å column (Phenomenex, Torrence, CA, USA), purity and identity were confirmed by RP-HPLC on a Jupiter 4 µm Proteo 90 Å C_12_ (Phenomenex), MALDI-ToF MS on an Ultraflex II and ESI MS on an HCT ESI (Bruker Daltonics, Billerica, MA, USA). RP-HPLC was performed employing linear gradients of eluent A (0.08% TFA in ACN) in eluent B (0.1% TFA in ACN).

### 2.3. Investigation of Plasma Stability

Investigation of peptide stability in blood plasma was carried out as described previously [22]. Tam-labeled peptides were dissolved in human blood plasma at a concentration of 10^−5^ M and incubated at 37 °C and 250 rpm. Samples taken at the respective time points were added to a solution of 0.1% SDS in ACN/EtOH (1:1). After incubation at −20 °C for 20 min, the supernatant was transferred to a new tube and incubated again at −20 °C for at least 3 h. The solution was filtered by centrifugation using Costar Spin-X tubes (0.22 µm), and the filtrate was analyzed by RP-HPLC on a VariTide RPC, 6 µm, 200 Å column (Agilent Technologies, Santa Clara, CA, USA) employing a linear gradient of 15–65% (*v*/*v*) A in B over 40 min. The fluorescence of the peptide was detected at λ = 573 nm. Peaks were integrated, and the peak containing the intact peptide was normalized to the sample taken at *t* = 0 min (100%). Plasma half-life was determined using one-phase decay in Prism 5 (GraphPad, San Diego, CA, USA).

### 2.4. Cell Culture

COS-7 and HEK293 cells were cultivated in DMEM supplemented with 10% FBS or DMEM/Ham’s F12 supplemented with 15% FBS, respectively. HMEC-1 cells were cultivated in MCDB131 with EGF (10 ng/mL) and hydrocortisone (1 µg/mL). All cells were maintained in T75 cell culture flasks at 37 °C, 95% humidity, and 5% CO_2_ (standard conditions).

### 2.5. Calcium Flux Assay

COS-7 cells were transfected in 75 cm^2^ cell culture flasks with 12 µg of the hCMKLR1_eYFP_G_αΔ6qi4myr__pV2 plasmid overnight using Metafectene Pro. Transfected cells were seeded in 96 well plates (100 µL cell suspension in DMEM + 10%FBS/well) and incubated overnight. The following day, the Ca^2+^- mobilization was performed as described previously [23]. Briefly, cells were incubated with Fluo-2-AM solution (2.3 µM Fluo-2-AM, 0.06% (*v*/*v*) Pluronic-F127 in assay buffer). After 1 h, the Fluo-2-am solution was replaced with assay buffer (20 mM HEPES, 2.5 mM Probenecid in HBSS, pH 7.5), and the basal Ca^2+^ level was measured for 20 s with a Flexstation 3 (λ_ex_ = 485 nm, λ_em_ = 525 nm). The ligand was added, and Ca^2+^-response was measured for 40 s. The resulting maximum over basal values was calculated for each well and normalized to the top and bottom values of the control curve (chemerin-9, **1**). All experiments were performed in triplicates, each experiment was repeated at least twice. Nonlinear regression was calculated using GraphPad Prism 5.

### 2.6. Bioluminescence Resonance Energy Transfer (BRET)

HEK293 cells were transiently transfected overnight with 7800 ng hCMKLR1-eYFP+ G_αΔ6qi4myr_ in pVitro2 and 200 ng Rluc8 Arrestin3 in 75 cm^2^ cell culture flasks using MetafectenePro according to the manufacturer’s protocol. One day post-transfection, cells were seeded in white 96 well plates coated with poly D-Lysine. Cells were again allowed to grow overnight under standard conditions and the assay was performed two days post-transfection. The medium was replaced with 100 µL BRET buffer (HBSS, 25 mM HEPES, pH 7.3), and 50 µL of the luciferase substrate coelenterazine-h (final concentration of 4.2 µM) was added. Cells were stimulated with peptides dissolved in BRET buffer, buffer without peptide was used as a negative control. The BRET signal was measured 15 min after stimulation with a Tecan infinite 2000 plate reader using two filter sets at 37 °C (luminescence filter 400–470 nm and fluorescence filter 505–590 nm) and the fluorescence/luminescence ratio was plotted as a function of peptide concentration. The values of the negative control were subtracted and nonlinear regression was calculated using GraphPad Prism 5. The curves were normalized to the top and bottom values of the type chemerin 9 curve (**1**). All measurements were performed in four technical replicates and all experiments were repeated at least twice.

### 2.7. Statistical Analysis

Activities of the peptides in the Ca^2+^ flux assay were compared employing a one-way ANOVA with Dunnett’s multiple comparison post test in GraphPad Prism 8, testing the pEC_50_ values from at least two independent experiments for each peptide. All information concerning technical and experimental replicates is given in the figure and table captions.

### 2.8. Fluorescence Microscopy

For fluorescence microscopy, HEK293 cells were seeded into Ibidi 15 µ-slides (140,000 cells/200 µL/well) coated with poly D-lysine and grown overnight. Next, cells were transfected with 900 ng CMKLR1-eYFP. Transfection was achieved using Lipofectamine 2000 according to the manufacturer’s protocol. One day post-transfection, fluorescence microscopy experiments were performed on an AxioVision Observer.Z1 microscope equipped with an ApoTome imaging system (Zeiss, Jena, Germany). Before the experiment, cells were starved in OptiMEM reduced serum medium containing Hoechst 33342 for 30 to 60 min. To observe peptide uptake, cells were stimulated with 1 µM Tam-chemerin-9 in OptiMEM, which was replaced with acidic wash (50 mM glycine, 100 mM NaCl, pH 3) in HBSS after the indicated time, followed by two washing steps with OptiMEM. Microscopy was carried out in OptiMEM.

### 2.9. Quantification of Peptide Uptake Using a High Content Imager

To quantify the receptor-mediated peptide uptake, the intracellular accumulation of Tam-fluorescence was observed at different time points. HEK293 cells stably transfected with CMKLR1-eYFP were seeded into 96 black well plates (100,000 cells/well) coated with poly D-lysine and incubated at 37 °C, 95% humidity, and 5% CO_2_ overnight. Prior to the experiment, cells were incubated with Hoechst 33342 in OptiMEM, which was replaced with pure OptiMEM after 30 min. Cells were stimulated with 1 µM of the respective, Tam-labeled peptide for the specified periods, followed by washing with acidic wash (50 mM glycine, 100 mM NaCl, pH 3) in HBSS. Microscopy was carried out in OptiMEM using an ImageXpress Micro Confocal High-Content Imaging System (Molecular Devices, San José, CA, USA), using the appropriate filters for the respective fluorophores (Table 1). The fluorescence intensity per cell was automatically analyzed for each well by a module detecting the nuclei (5–30 µm in diameter and 100 gray levels above background) and the granules by Tam-peptide fluorescence (2–5 µm in diameter, 70 gray levels above background).

### 2.10. Resazurin Assay

HEK293 cells stably transfected with CMKLR1-eYFP, empty HEK293 cells (15,000 cells in 100 µL suspension/well), or HMEC-1 cells (5000 cells in 100 µL suspension/well) in their respective cell culture media were seeded in 96 well plates (coated with poly D-lysine for HEK cells) and incubated at standard conditions overnight. Afterward, cells were incubated with peptides or MTX dissolved in cell culture media, and a medium without peptides was used as a control. After 72 h, cells of the negative control were treated with ethanol for 3 min. Ethanol or media was removed and a 50 µL resazurin-based in vitro toxicology assay kit (Sigma-Aldrich, St. Lous, MO, USA) in cell culture media (1:10) was added to each well. After 1 h incubation time, the conversion of the resazurin was detected by measuring the fluorescence intensity at λ_em_ = 590 nm. All experiments were performed in duplicates and each experiment was repeated at least three times. Nonlinear regression was calculated using GraphPad Prism 5, curves were normalized to untreated and ethanol-treated cells.

## 3. Results

### 3.1. Set-Up to Investigate Stabilized Chemerin Analogues

**Figure 1:**
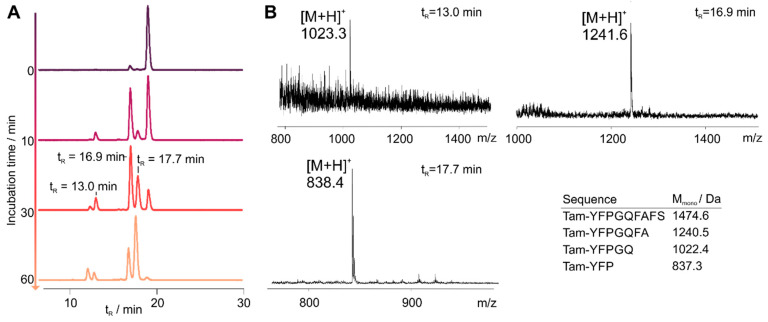
Stability of Tam-chemerin-9 (**1T**) in human blood plasma. (**A**) RP-HPLC traces of samples Table 573 nm. (**B**) MALDI ToF mass spectrometry analysis of samples taken from the peaks depicted in (**A**).

In order to investigate the stability of chemerin-9 in human blood plasma, we synthesized an N-terminally 6-carboxytetramethylrhodamin (Tam)-labeled chemerin-9 analog (**1T**). Sequence and analytical characterization are displayed in Table 2 and Table 3, respectively. After incubation in blood plasma at 37 °C, we took samples at distinct time points and analyzed them by RP-HPLC (Figure 1A). As expected, the peptide is rapidly degraded, and less than 1% intact peptide is detected after 1 h incubation. We collected fractions of the peaks after 30 min and analyzed them by MALDI ToF MS to identify the most critical cleavage sites (Figure 1B). The first dominant degradation product at a retention time (t_R_) of 16.9 min corresponds to the cleavage after A^7^, identified by its corresponding (M + H)^+^ signal at m/z = 1241.6 (M_mono_ = 1240.5 Da). A less prominent fragment is eluted at t_R_ = 13.0 min and corresponds to cleavage after Q^5^. This fragment is also identified by the (M + H)^+^ signal at m/z = 1023.3 (M_mono_ = 1022.4 Da). The degradation product that becomes most prominent after 1 h incubation corresponds to cleavage after P^3^, and is the smallest fragment that was detected in our assay set-up.

### 3.2. Cyclization Prevents C-Terminal Degradation

**Figure 2:**
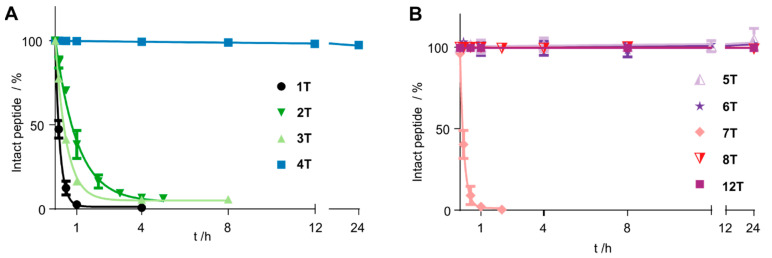
Stability of Tam-modified cyclic chemerin-9 derivatives in blood plasma. Data points represent mean ± SEM values from at least two independent experiments. (**A**) Cyclization through positions 4 and 7 only slightly improves stability, regardless of the cyclization method (disulfide in **2T**, thioacetal in **3T**). Including the C-terminal amino acids in the ring by cyclization through positions 4 and 9 gives peptide **4T**, which is not degraded over the course of the experiment. (**B**) Changing the method of cyclization in peptides cyclized through positions 4 and 9 does not alter plasma stability (**5T**, **6T**). A free N-terminus proves susceptible to degradation (**7T**), but introducing an N-terminal D-Tyr restores stability (**12T**).

To stabilize the peptide, we introduced cyclization at chosen positions. Since the peptide bond between A^7^ and F^8^ was susceptible to proteolytic cleavage, we replaced the residues with cysteine at positions 7 or 9, respectively. As all aromatic residues are crucial for receptor binding and activation, we additionally exchanged G^4^ by D-cysteine, which retained activity [10]. All sequences of the Tam-labelled peptides are displayed in Table 2 and Table 3. The resulting disulfide **2T** displayed only slightly increased stability, and even replacing the disulfide bond for a thioacetal in peptide **3T** did not improve its resistance to proteolytic degradation (Figure 2). In contrast, the cyclic disulfide **4T** displayed superior stability and remained completely intact for the course of the experiment (24 h). As expected, derivatives of this peptide in which we replaced the disulfide by a thioacetal (**5T**) and, additionally, the C-terminal cysteine by homocysteine (HCys, **6T**), demonstrated the same stability for 24 h (Table 2 and Figure 2).

### 3.3. N-Terminal Modification Yields Metabolically Stable Peptides

**Figure 3:**
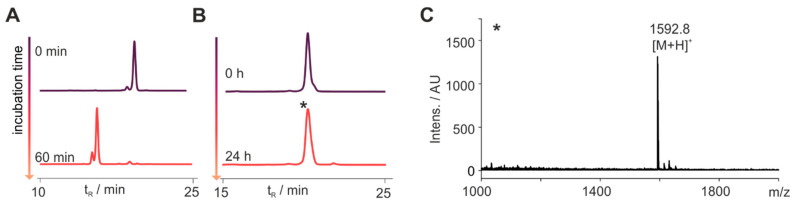
N-Terminal modification is essential for metabolic stability. (**A**) Bearing a free N-terminus, the cyclic peptide **7T** is fully degraded after 60 min. (**B**) Introducing a D-Tyr in position 1 yields the metabolically stable peptide **8T**, which is not degraded for 24 h in blood plasma. RP-HPLC traces of samples are displayed taken at the indicated time points detecting the fluorescence at λ_em_ = 573 nm. (**C**) Mass spectrometrical analysis of the peak indicated by an asterisk (*) confirmed the integrity of the peptide **8T** after 24 h through its (M + H)^+^ signal at *m*/*z* = 1592.8 (M_mono_ = 1591.6).

The N-terminus of the peptides **1**–**6T** was modified with a Tam-moiety, which we suspected to prevent N-terminal degradation. Thus, we synthesized the cyclic disulfide **7T** where the Tam-label was introduced through a lysine side chain in position 7. Indeed, this peptide was completely degraded within 60 min (Figure 2 and Figure 3). However, simply introducing a D-Tyr at position 1 resulted in a peptide that was fully stable in blood plasma for 24 h (**8T**, Figure 3B). To verify the integrity of the peptide after 24 h, we collected a sample of the peak detected by RP-HPLC and analyzed it by MALDI ToF MS. We exclusively detected the (M + H)^+^ signal of the intact peptide at *m*/*z* = 1592.8 (M_mono_ = 1591.6), confirming that no degradation occurred over the course of the experiment (Figure 3C).

### 3.4. Stabilized Chemerin-9 Analogs Activate CMKLR1

**Figure 4:**
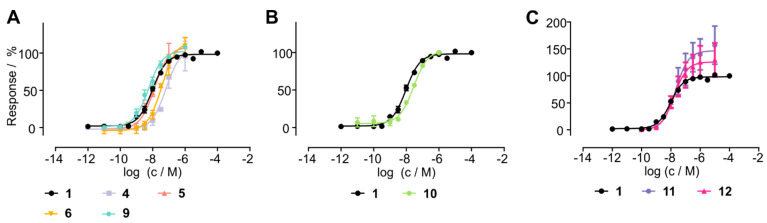
Activity of cyclic chemerin-9 derivatives tested in a Ca^2+^ flux assay. (**A**) Influence of different side chain cyclization on activity, whereas cyclization through D-Hcys^4^ and Cys^9^ yields the most active peptide **9**. (**B**) N-Terminal stabilization by a D-Tyr preserves activity of the cyclic peptide **10**. (**C**) N-Terminal attachment of either an EG(4) linker (**11**) or a GFLG linker (**12**) does not impact activity. All data points represent mean ± SEM of at least two independent experiments performed in triplicates.

To test whether metabolically stable cyclic analogs of chemerin-9 retain biological activity, we tested the peptides in a Ca^2+^ assay for their ability to activate CMKLR1 (Figure 4, Table 4). First, we examined the influence of different cycles on the activity by comparing peptides cyclized between positions 4 and 9 (Figure 4A). With an EC_50_ of 64 nM, the cyclic disulfide **4** displayed a significant (*p* < 0.0001, one-way ANOVA with Dunnett’s multiple comparison post-test) loss of activity compared to the linear peptide **1**. With an EC_50_ = 13 nM, the corresponding thioacetal **5** was as active as the linear peptide. Simultaneously introducing a thioacetal and a Hcys in position 9 yielded peptide **6**, which was again slightly less active than the wild-type peptide **1** (EC_50_ = 37 nM, *p* = 0.025), while exchanging position 4 for D-Hcys yielded analog **9**, which was as active as **1** (EC_50_ = 5 nM).

As N-terminal modification is necessary for metabolic stability, we investigated whether an N-terminal D-Tyr is tolerated for receptor activation as described previously [24]. We synthesized analog **10** which displayed an EC_50_ of 28 nM and was as active as the corresponding L-Tyr-bearing derivative **6**. N-terminal modification with either the cleavable GFLG linker (analog **11**) or ethylene glycol (peptide **12**) retained activity, demonstrating that N-terminal attachment of a linker does not reduce the potency of the analogs.

### 3.5. Cyclic Chemerin-9 Derivatives Are Suitable for Intracellular Delivery

**Figure 5:**
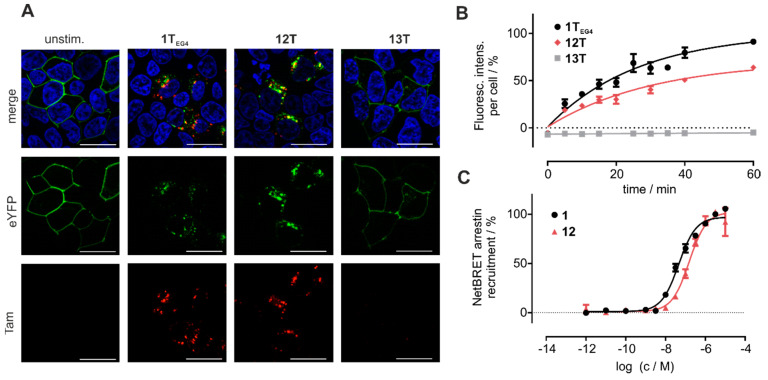
Cyclic chemerin-9 is internalized by cells expressing CMKLR1. (**A**) Live cell fluorescence microscopy of HEK293 cells transiently transfected with CMKLR1-eYFP. Cells were incubated with 1 µM of the respective, Tam-labeled peptide for 60 min. Intracellular Tam-fluorescence is visible after stimulation with the linear peptide **1T_EG4_** and the cyclic analog **12T**, but not for the (A^8^) mutant **13T**. Representative images from at least two independently performed experiments are displayed. Scale bar = 10 μM, 63× magnification (**B**) The uptake of Tam-labeled peptides was quantified by measuring the intracellular accumulation of Tam-fluorescence in HEK293 cells stably transfected with CMKLR1-eYFP using a High Content Imager. The uptake of the cyclic peptide **12T** is comparable to the linear peptide **1T_EG4_**, while **13T** is not internalized over time. Data points represent mean ± SEM from at least two independent experiments performed in duplicates. (**C**) The linear peptide **1** and the cyclic derivative **12** induce arrestin3 recruitment to CMKLR1 as measured by a BRET-based arrestin-recruitment assay. Data points represent mean ± SEM from at least two independent experiments performed in quadruplicates.

Chemerin may be an attractive shuttling system to selectively target cancer tissue overexpressing CMKLR1. To investigate whether cyclic chemerin-9 derivatives are suitable to shuttle cargo into the cell, we set up live-cell fluorescence microscopy experiments in HEK293 cells transiently transfected with CMKLR1 (Figure 5A). After 60 min stimulation with the fluorescent linear analog **1T_EG4_**, the receptor is internalized and co-localizes with intracellular Tam-fluorescence, indicating that the peptide **1T_EG4_** has been shuttled into the cell. The same observation is made for the cyclic derivative **12T**, where pronounced intracellular fluorescence is visible after 60 min of stimulation. 

To quantify the uptake of Tam-labeled peptides in a time-resolved fashion, HEK293 cells stably transfected with CMKLR1-eYFP were seeded in 96 well plates, incubated with 1 µM of the respective ligands, and washed after periods of up to 1 h. The cells were then analyzed using an ImageXpress Micro Confocal High-Content Imaging System (Molecular Devices, St. Louis, MO, USA). The linear peptide **1T_EG4_,** as well as the cyclic derivative **12T**, show comparable intracellular accumulation over time, confirming the results obtained in live-cell microscopy (Figure 5). The alanine-substituted Tam-labeled linear peptide Tam-EG(4)-(A^8^)-chemerin-9 **13T** served as a negative control, and did not accumulate in the cells (Figure 5A,B).

To characterize the concentration-dependent internalization of CMKLR1-eYFP, we employed a BRET-based arrestin3-recruitment assay. Because the most common fluorophores, Tam and carboxyfluorescein distort the signal in this set-up, a peptide bearing an EG(4)-linker, but no fluorophore was tested (**12**). Peptide **1** displayed an EC_50_ of 43 nM, and the activity of **12** was slightly reduced in comparison (EC_50_ = 154 nM).

**Figure 6:**
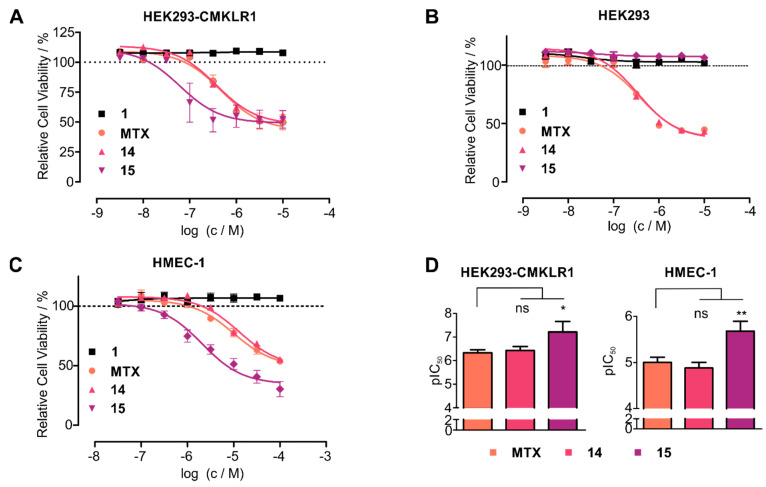
Cyclic chemerin-9 derivatives can shuttle MTX into CMKLR1-expressing cells. (**A**) Toxicity of MTX and MTX-modified chemerin-9 analogs on stably transfected HEK293-CMKLR1 were tested in a resazurin-based cell viability assay. Chemerin-9 (**1**) itself is not toxic, MTX and the MTX-modified linear peptide **14** demonstrate moderate toxicity. In contrast, the stabilized MTX-modified analog **15** demonstrates a significantly higher potency. (**B**) Toxicity of MTX and MTX-peptide conjugates on untransfected HEK293 cells. The stabilized conjugate **15** is not toxic at concentrations up to 10 µM, confirming the receptor-dependent toxicity of this conjugate. (**C**) Analog **15** displays superior toxicity compared to MTX alone or the linear conjugate **14** on endogenous CMKLR1-expressing HMEC-1 cells. Data points represent mean ± SEM from at least three independent experiments performed in duplicates. (**D**) Statistical analysis of pIC_50_ values of MTX and analogs **14** and **15** on HEK293-CMKLR1 and HMEC-1 cells, bars represent mean ± SD. On both cell lines, **15** is significantly more toxic than free MTX (* *p* < 0.05, ** *p* < 0.01, ns: not significant, *p* > 0.05 in a one-way ANOVA with Dunnett’s post test).

Thus, we hypothesized that stabilized chemerin-9 analogs can be used to selectively shuttle toxophores such as methotrexate (MTX) into CMKLR1-expressing cells. Therefore, we attached MTX through the cleavable GFLG linker either to linear chemerin-9 yielding analog **14** or to a stable cyclic derivative (**15**). As MTX contains two carboxy-groups available for coupling, this resulted in an isomeric mixture of α- and γ-MTX-derivatives for both peptides. To investigate the toxicity of the conjugates, we set up resazurin-based cell viability assays with HEK293 cells stably transfected with CMKLR1-eYFP (Figure 6A). As expected, chemerin-9 (**1**) itself did not impact cell viability. With an IC_50_ of 471 nM, MTX itself displayed moderate toxicity, similar to the linear, MTX-modified peptide **14** (IC_50_ = 347 nM). Strikingly, the stabilized, MTX-modified cyclic peptide **15** demonstrated an almost eightfold increase in toxicity (IC_50_ = 61 nM) compared to MTX itself. In contrast, analog **15** exerted no toxicity on HEK293 cells devoid of CMKLR1, demonstrating that the toxic effect of this conjugate is receptor-dependent (Figure 6B). As observed for HEK293-CMKLR1 cells, the linear MTX-modified peptide **14** displayed an IC_50_ similar to free MTX (IC_50_ = 345 nM and IC_50_ = 373 nM, respectively).

To test whether cells endogenously expressing CMKLR1 have sufficient levels of receptor for efficient chemerin-mediated drug shuttling, we tested the toxicity of our conjugates on HMEC-1 cells [25] (Figure 6C). Again, as for transfected HEK293 cells, conjugate **15** demonstrated superior toxicity (IC_50_ = 2 μM) compared to free MTX (IC_50_ = 10 μM) or the linear peptide **14** (IC_50_ = 13 µM). On both CMKLR1-expressing cell lines (HEK293-CMKLR1 and HMEC-1), analog 15 was significantly more toxic than free MTX (Figure 6D). These results demonstrate that cyclic chemerin-9 derivatives are suitable to be used in peptide–drug conjugates that specifically deliver toxophores into cells expressing CMKLR1.

## 4. Discussion

Chemerin was initially identified as an inflammatory protein in ascitic fluids [2]. It induces the migration of dendritic cells, natural killer (NK) cells, and macrophages to sites of inflammation, and consequently has gained significant interest for the treatment of inflammatory disorders [2,12]. However, the design of small molecules targeting the chemerin receptor CMKLR1 was of limited success so far, although some compounds have made it to clinical trials in the past years [26]. Until now, harnessing the therapeutic potential of chemerin itself was limited by the difficult production of the full-length protein and the low plasma stability of the C-terminal peptide chemerin-9 [27].

First, we investigated the degradation of chemerin-9 in blood plasma to identify the most crucial positions for stabilization. We determined a plasma half-life of 10 min, which is similar to the previously reported half-life of 24 min [24]. The first, rapidly arising degradation product is formed by cleavage after A^7^, equivalent to the formation of the inactive chemerin species chemerin^21−155^ in vivo [8]. We previously showed that chemerin-9 activates the CMKLR1 adopting a turn conformation, which prompted us to employ cyclization to stabilize the peptide [10]. As all aromatic residues are crucial for receptor binding and activation, we synthesized cyclic analogs by introducing D-Cys in position 4 and Cys in positions 7 or 9, respectively [9]. Cyclization through positions 4 and 9 yielded peptides that were stable in blood plasma, independent of the exact method of ring closure: D-Cys to Cys by a disulfide or a thioacetal (**4T**, **5T**), D-Cys to Hcys through a thioacetal (**6T**), or D-Hcys to Cys through a disulfide (**12T**). N-terminal degradation of the peptide can easily be prevented by either introducing an N-terminal D-Tyr (**8T**) or by attaching a linker, e.g., ethylene glycol (**12T**). Therefore, all peptides presented here that contain an N-terminal modification and a cyclization through positions 4 and 9, are not degraded in blood plasma for at least 24 h. Thus, they demonstrate higher stability than previously designed chemerin-9 analogs with a plasma half-life of 4 h or 28 h, respectively [24,28].

It is evident that the metabolically stable peptides need to retain biological activity in order to be useful for therapeutic applications. Therefore, we tested the stable cyclic peptides in a Ca^2+^ flux assay to examine the impact of the modifications on the potency of the peptides. Indeed, the disulfide **4** was significantly less active than linear chemerin-9 (**1**). However, simply exchanging position 4 to D-Hcys yielded the highly active analog **5** [10]. N-terminal modification with D-Tyr is well tolerated (**10**), and attaching ethylene glycol (**12**) or the cathepsin B cleavable GFLG linker [29] (**11**) still results in cyclic peptides that are as active as the unmodified linear peptide **1**.

By fluorescence microscopy and BRET assays, we demonstrate that cyclic derivatives of chemerin-9 induce receptor internalization and are shuttled into CMKLR1-expressing cells in a short time. This can be used for intracellular delivery of toxophores such as MTX into the cells. While the MTX-modified linear peptide **14** displays the same toxicity as MTX itself, the toxicity of the stabilized MTX-modified analog **15** is significantly increased. We hypothesize that **14** is rapidly degraded and thus releases the toxophore outside of the cell, which explains the similar toxicity as MTX itself. The superior toxicity of analog **15** is preserved towards HMEC-1 cells, which endogenously express CMKLR1. In contrast, **15** displays no toxicity towards untransfected HEK293 cells, demonstrating that this analog can be used to selectively shuttle toxophores into CMKLR1-expressing cells.

Recently, chemerin has gained increasing interest regarding its role in cancer, and competing roles have been proposed for chemerin in the context of tumor progression [27]. Downregulation of local chemerin levels seems to be part of an immune evasion strategy of different cancer types. Chemerin mRNA levels are significantly lower in breast cancer tissues compared to surrounding healthy tissue, and forced overexpression of chemerin leads to infiltration of the tumor microenvironment with NK cells, dendritic cells, and macrophages, thereby suppressing tumor growth [30]. A similar role was proposed in melanoma, where chemerin-mediated NK cell recruitment to the tumor microenvironment suppressed metastasis [31]. In hepatocellular carcinoma, downregulation of chemerin correlates with poor patient prognosis, and administration of chemerin in mice reduced metastazation [32]. In line with these results, overexpression of chemerin in mice decreased the number of metastases in a model of hepatocellular carcinoma [33]. Therefore, administration of stabilized chemerin-derived peptides might be an attractive approach for cancer immunotherapy, an emerging concept which aims at counteracting the immune evasion strategies adopted by many tumors [34]. However, a downside of this approach is that it might be necessary to inject chemerin-derived peptides directly into the tumor or in its vicinity.

While chemerin is frequently downregulated in several cancer types, the chemerin receptor CMKLR1 is directly involved in the progression of some tumors. In neuroblastoma, high CMKLR1 expression correlates with decreased survival and blocking CMKLR1 reduced cell viability of neuroblastoma cells in vitro and tumor progression in vivo [35]. Colorectal cancer tissue displays increased levels of CMKLR1, correlating with tumor size [36]. Expression of CMKLR1 was also demonstrated in oesophageal squamous cancer cells, and activation of CMKLR1 promoted the invasion of these cells in vitro [15].

In a recent study, Erdmann et al. used chemerin-derived peptides as a carrier for a PET-tracer, successfully visualizing CMKLR1-positive breast cancer xenografts in mice [37]. These results demonstrate that it is possible to selectively address cancer tissue by targeting CMKLR1. This is the prerequisite for cancer therapy employing peptide-drug conjugates (PDC), an emerging concept that has gained increasing interest in recent years. Activating their cognate GPCR through the peptide part, PDCs are co-internalized together with the receptor, delivering their toxic cargo into the cell. Therefore, the toxicity of the cargo is combined with the target selectivity of the peptide ligand, reducing side effects and potentially increasing the therapeutic window [38]. This strategy was previously employed to overcome drug resistance in breast cancer cells [39]. It can be used to deliver various cargoes into the cells, such as carboranes for boron neutron capture therapy, or even transcription factors [40,41]. Using an enzyme-cleavable linker like the GFLG sequence can ensure the intracellular release of the toxic payload [29]. Our results suggest that the potency of chemerin-9 is robust towards N-terminal modification, allowing for various linker structures or toxophores to be attached as demonstrated here for MTX.

## 5. Conclusions

Taken together, we present a series of metabolically stable cyclic peptides based on chemerin-9. Cyclization through positions 4 and 9 can be combined with different N-terminal modifications such as D-amino acids, EG(4), or a cleavable linker to yield a stable peptide, offering a flexible platform for the choice of cyclic chemerin-9 analogs. We demonstrate that these peptides are suitable for specific delivery of toxic small molecule cargoes into cells, opening the path towards specific PDC for CMKLR1-expressing tumors. Chemerin has recently started to show great potential for the treatment of various cancer types but is challenging to produce. Hence, our peptides represent highly active, metabolically stable molecules with well-characterized structure–activity relationships. They are synthetically accessible, can be modified according to their desired mode of action, and thus represent an important step towards targeting CMKLR1 in cancer therapy.

## Figures and Tables

**Table 1:** Filter sets used for fluorescence microscopy.

Fluorophore	Used for Labeling	Excitation (nm)	Emission (nm)
Hoechst33342	cell nucleus	352	455
Tam	peptide	549	577
eYFP	CMKLR1	514	526

Hoechst33342: 2′-(4-Ethoxyphenyl)-6-(4-methyl-1-piperazinyl)-1*H*,3′*H*-2,5′-bisbenzimidazole; YFP: yellow fluorescent protein; CMKLR1: chemokine-like receptor 1.

**Table 2:** Plasma stability of Tam-modified chemerin-9 derivatives determined in at least two independent experiments. Half-life t_1/2_ was determined in GraphPad Prism with one-phase decay. Sites of cyclization are indicated by square brackets. Substitution of disulfides by methylene thioacetals is marked by a methylene group in the sequence. x = D-homocysteine.

Compound Number	Sequence	t_1/2_/h
**1T**	Tam-YFPGQFAFS	<0.2
**2T**	Tam-YFP(cQFC)FS	0.75
**3T**	Tam- YFP(c(CH_2_)QFC)FS	0.3
**4T**	Tam-YFP(cQFAFC)	>24
**5T**	Tam-YFP(c(CH_2_)QFAFC)	>24
**6T**	Tam-YFP(c(CH_2_)QFAFX)	>24
**7T**	YFP(cQFK(Tam)FC)	<0.2
**8T**	yFP(cQFK(Tam)FC)	>24
**12T**	Tam-EG(4)-YFP(xQFAFC)	>48 h

**Table 3:** Analytical data of the investigated peptides. Square brackets indicate sites of cyclization by disulfide bonds or thioacetals. Methyl groups in the sequence indicate a thioacetal between the two side-chain sulfur atoms in the respective peptide.

Compound Number	Sequence	M_mono_	M_obs_	t_R_/%B	t_R_/%B	Purity
**1**	YFPGQFAFS	1062.5	1063.5	41.7 ^a^	30.2 ^b^	>95
**1T**	Tam-YFPGQFAFS	1474.6	1475.6	50.5 ^a^	43.8 ^c^	>95
**1T_EG4_**	Tam-EG(4)-YFPGQFAFS	1721.7	1722.7	51.0 ^a^	40.5 ^b^	>95
**2T**	Tam-YFP(cQFCFS)	1550.6	1551.6	54.2 ^a^	48.4 ^c^	>95
**3T**	Tam-YFP(c(CH_2_)QFCFS)	1564.6	1565.5	53.8 ^a^	48.5 ^c^	>95
**4**	YFP(cQFAFC)	1122.4	1123.4	46.5 ^a^	34.2 ^b^	>95
**4T**	Tam-YFP(cQFAFC)	1534.6	1535.6	54.4 ^a^	47.9 ^c^	>95
**5**	YFP(c(CH_2_)QFAFC)	1136.5	1137.5	45.5 ^a^	36.0 ^c^	>95
**5T**	Tam-YFP(c(CH_2_)QFAFC)	1548.6	1549.6	53.6 ^a^	48.2 ^c^	>95
**6**	YFP(c(CH_2_)QFAFX)	1150.5	1151.5	46.1 ^a^	37.4 ^b^	>95
**6T**	Tam-YFP(c(CH_2_)QFAFX)	1562.6	1563.6	55.0 ^a^	43.9 ^b^	>95
**7T**	YFP(cQFK(Tam)FC)	1591.6	1592.6	49.1 ^a^	42.6 ^c^	>95
**8T**	yFP(cQFK(Tam)FC)	1591.6	1592.7	50.1 ^a^	15.9 ^b^	>95
**9**	YFP(xQFAFC)	1136.4	1137.5	45.6 ^a^	34.3 ^b^	>95
**10**	yFP(c(CH_2_)QFAFX)	1150.5	1151.4	47.9 ^a^	36.1 ^b^	>95
**11**	GFLGYFP(xQFAFC)	1552.6	1553.6	48.1 ^a^	37.6 ^b^	>95
**12**	EG(4)-YFP(xQFAFC)	1383.6	1384.6	47.4 ^a^	37.4 ^b^	>95
**12T**	Tam-EG(4)-YFP(xQFAFC)	1795.7	1796.7	48.6 ^a^	38.6 ^b^	>95
**13T**	Tam-EG(4)-YFPGQFAAS	1233.6	1234.6	47.5 ^a^	36.8 ^b^	>95
**14**	Mtx-GFLGK(Ac)YFPGQFAFS	2042.9	2043.9	42.8/43.1 ^a^	38.3/38.6 ^b^	>95
**15**	Mtx-GFLGK(Ac)YFP(xQFAFC)	2116.9	2117.9	39.4/40 ^a^	35/35.6 ^b^	>95

t_R_: Elution in RP-HPLC with a linear gradient of 20–70% B in A over 40 min with ^a^ flow rate of 1.0 mL/min on a Jupiter 4 µm Proteo 90 Å C_12_ column, ^b^ flow rate 1.55 mL/min on an Aeris 3.6 µm 100 Å XB-C18 column, or ^c^ flow rate 1.55 mL/min on a Kinetex 5 µm biphenyl 100 Å column. TA: Thioacetal; X: Homocysteine; Tam: 6-carboxy tetramethylrhodamine. EG(4): polyethylene glycol with four ethylene glycol units.

**Table 4:** Activity of chemerin-9 derivatives. All data were obtained from at least two independent experiments performed in triplicates (Ca^2+^ flux) or quadruplicates (arrestin recruitment). Data were normalized to the top and bottom values of the chemerin-9 curve, and nonlinear regression was performed with a three-parameter curve fit in GraphPad Prism 5. X = Homocysteine, x = D-homcysteine.

Compound Number	Sequence	EC_50_/nM	pEC_50_ ± SEM
Ca^2+^ Flux			
**1**	YFPGQFAFS	10	8.021 ± 0.04
**4**	YFP(cQFAFC)	64	7.192 ± 0.15
**5**	YFP(c(CH_2_)QFAFC)	13	7.888 ± 0.13
**6**	YFP(c(CH_2_)QFAFX)	37	7.429 ± 0.11
**9**	YFP(xQFAFC)	5	8.259 ± 0.07
**10**	yFP(c(CH_2_)QFAFX)	28	7.553 ± 0.07
**11**	GFLGYFP(xQFAFC)	21	7.676 ± 0.22
**12**	EG(4)-YFP(xQFAFC)	17	7.766 ± 0.23
Arrestin3 Recruitment			
**1**	YFPGQFAFS	46	7.338 ± 0.04
**12**	EG(4)-YFP(xQFAFC)	154	6.813 ± 0.06

TA: Thioacetal; X: Homocysteine; Tam: 6-carboxy tetramethylrhodamine. EG(4): polyethylene glycol with four ethylene glycol units.

## Data Availability

No new data were created or analyzed in this study. Data sharing is not applicable to this article.
